# Experiences of Spanish out-of-hospital emergency workers with high levels of depression during the COVID-19 pandemic: a qualitative study

**DOI:** 10.1186/s13690-023-01233-w

**Published:** 2024-01-30

**Authors:** Susana Navalpotro-Pascual, María Paz Matellán-Hernández, Elena Pastor-Benito, Raúl Soto-Cámara, Rosa M Cárdaba-García, Noemi García-Santa-Basilia, Henar Onrubia-Baticón, Vinita Mahtani-Chugani

**Affiliations:** 1 Nursing Department, Faculty of Medicine, Autonomous University of Madrid, Madrid, Spain; 2 Emergency Medical Service of Madrid-SUMMA 112, Madrid, Spain; 3 Prehospital Emergency Research Network (RINVEMER), Spanish Society of Emergency Medicine (SEMES), Madrid, Spain; 4 Emergency Medical Service of Castilla and León-Sacyl, Valladolid, Spain; 5 Department of Health Sciences, University of Burgos, Burgos, Spain; 6 Faculty of Nursing of Valladolid, Valladolid, Spain; 7 Nursing Care Research Group (GICE), Department of Nursing, University of Valladolid, Valladolid, Spain; 8 Healthcare Quality Assessment and Information Systems Service, Santa Cruz de Tenerife, Tenerife, Spain; 9 Primary Care Management of Tenerife, Santa Cruz de Tenerife, Tenerife, Spain

**Keywords:** COVID-19, Health personnel, Emergency medical system, Qualitative research, Mental health, COVID-19, Personal sanitario, Sistema de emergencias médicas, Investigación cualitativa, Salud mental

## Abstract

**Background:**

The COVID-19 pandemic had a major psychological impact on health care workers (HCWs). This study was embedded in a larger quantitative study on the mental health care of out-of-hospital HCWs in Spain. To better understand this, a qualitative study was conducted to explore the experiences, coping strategies, and influencing factors of out-of-hospital HCWs who scored high (> 25 points) on the Depression Anxiety Stress Scale (DASS-21) in a previous quantitative mental health study.

**Methods:**

A qualitative study was conducted using six in-depth interviews with individuals who scored high on the depression scale and agreed to be contacted by email between May and June 2021, using the phenomenological approach. The data were analysed using Brawn and Clare’s method.

**Results:**

The main results findings related to four themes. The emotional impact of assuming a professional role with high self-demands and responsibilities; Factors influencing the development of negative emotions such as the cruelty of the pandemic, the helplessness in relation to health management and policy, the changing role of the professional and the relationship with society; Personal protection through coping strategies to manage negative emotions such as support from colleagues and family; Good practices for the future looking for different management strategies that can influence individuals and their personal, professional, and social relationships.

**Conclusion:**

The strong impact of the circumstances experienced points to the need to develop psychological support programmes that can protect people’s mental health from depression during a crisis and improve the relationship between workers and their managers.

**Supplementary Information:**

The online version contains supplementary material available at 10.1186/s13690-023-01233-w.

## Resumen

**Antecedentes:**

La pandemia de COVID-19 tuvo un gran impacto psicológico en el personal sanitario. Este estudio forma parte de un estudio cuantitativo más amplio sobre la atención a la salud mental de los trabajadores sanitarios extrahospitalarios en España. Para comprenderlo mejor, se llevó a cabo un estudio cualitativo para explorar las experiencias, estrategias de afrontamiento y factores de influencia de los trabajadores extrahospitalarios que obtuvieron puntuaciones altas (> 25 puntos) en la Escala de Depresión, Ansiedad y Estrés (DASS-21) en un estudio cuantitativo previo sobre salud mental.

**Métodos:**

Se realizó un estudio cualitativo mediante seis entrevistas en profundidad con individuos que puntuaron alto en la escala de depresión y aceptaron ser contactados por correo electrónico entre mayo y junio de 2021, utilizando el enfoque fenomenológico. Los datos se analizaron utilizando el método de Brawn y Clare.

**Resultados:**

Los principales hallazgos de los resultados se relacionaron con cuatro temas. El impacto emocional de asumir un rol profesional con altas autoexigencias y responsabilidades; Factores que influyen en el desarrollo de emociones negativas como la crueldad de la pandemia, la impotencia en relación con la gestión y la política sanitaria, el papel cambiante del profesional y la relación con la sociedad; Protección personal a través de estrategias de afrontamiento para gestionar las emociones negativas como el apoyo de los compañeros y la familia; Buenas prácticas para el futuro buscando diferentes estrategias de gestión que puedan influir en los individuos y en sus relaciones personales, profesionales y sociales.

**Conclusión:**

El fuerte impacto de las circunstancias vividas apunta a la necesidad de desarrollar programas de apoyo psicológico que puedan proteger la salud mental de las personas durante una crisis y mejorar la relación entre los trabajadores y sus jefes.

**Table:** 

**Text box 1.** **Contributions to the literature**
– Healthcare workers in out-of-hospital emergencies have been psychologically affected by the COVID19 pandemic, with high levels of depression in some cases. Are the causes the same as in other groups? Have Emergency Medical Technicians (EMTs) been studied in the same way as other groups?
– Experiences outside the hospital have been different, as the conditions and development of the activity have had to be adapted to the real situation
– Individualized preventive programmes of psychological support in times of crisis, knowing their most vulnerable points from experience of other crises, can be an intelligent strategy that facilitates less psychological deterioration and fatigue among professionals, as well as better patient care

## Background

COVID has been the first great pandemic of the twenty-first century [1] and has had serious economic, social, political, and cultural consequences for people at an individual and collective levels around the world [2].

In December 2019, a new disease appeared emerged from Wuhan, Hubei Province of China, with cases of atypical pneumonia with no known cause, later confirmed to be as a betacoronavirus (RNA) with similar characteristics to the Severe Acute Respiratory Syndrome Corona-Virus 1 (SARS-COV-1), named SAR-COV-2 [3]. The rapid spread of the virus between continents led the World Health Organisation (WHO) to declare the disease "the sixth public health emergency of international concern" beginning with the emergence of the pandemic situation on 11 March 2020″. At that time, 114 countries were already affected, with 118 confirmed cases and 4291 deaths [4]. In Spain, the cumulative confirmed COVID 19 cases from 19 January 2020 to 1 February 2021 was 2.86 million [1]. During the COVID-19 pandemic, frontline professionals paid dearly for their mental health, with the prevalence of depression among frontline clinical nurses who went to assisted Wuhan City being 20–47.1% [5].

The emergence of this pandemic put the health system to the test, revealing its capacities and weaknesses [6]. Health Care Workers (HCWs) were among those most affected by the pandemic. In particular, those who provided direct care to patients and were most at risk of infection were severely affected [7]. Frontline HCWs experienced physical and mental exhaustion, high levels of anxiety and depression, and other emotional disturbances, such as cognitive dysfunctional reactions, sleep problems, difficulty in interpersonal relationships, substance use behaviours, and even post-traumatic stress [8]. Several researchers reported different causal factors for this effect. They were exposed to high workloads during long working hours, with significantly reduced rest periods, lack of approved personal protective equipment and lack of clear and defined protocols for action; all of these circumstances increased their risk of infection [9, 10]. Other factors were an increase in the workload [11], the lack of protective equipment [12], the fear of infecting their family and friends, suffering isolation, and social discrimination [13], as well as, seeing that the patients they assisted were alone and died because of COVID-19 [14]. In addition, according to the Bueno-Ferrán study, female health professionals were the ones who most frequently developed symptoms of stress, anxiety, and depression during the pandemic [15]. Women presented higher levels of stress and anxiety related to caring for COVID-19 patients. They also more frequently developed post-traumatic stress associated with their work as healthcare workers [10]. In other studies, also carried out in Spain, concluded that the mental well-being of frontline health workers was affected during pandemics, with medium to high levels of anxiety, depression, nervousness, and insomnia, and to a lesser extent stress, specially of the EMTs [9, 10]. It is also noted that the media was a major source of increased stress and anxiety among the public [12].

Emergency medical services (EMS) personnel are tasked with providing immediate medical care to people outside the hospital, unlike hospital staff who work in a controlled environment. In the out-of-hospital setting, care is provided in private homes or on the street, and these professionals are exposed to the weather conditions of the moment. In addition, the resources available in an ambulance or helicopter are more limited than in a hospital. There is also the risk of higher than usual air or road speeds and exposure to road traffic accidents, which can increase anxiety levels [10]. It should be noted that this group of workers is regularly exposed to violence, aggression, and traumatic situations [16], which makes them more vulnerable to mental health problems than other healthcare workers [17–19]. However, during the pandemic they had to assist and transport many severely depressed patients from home, and experienced intense anxiety and stress on each call [9]. Their experiences and stories are a rich source of knowledge to assess the problem in depth.

As early as September 2020, Nelson and Kaminsky [20] pointed out that health workers will suffer psychological burnout in this pandemic, given the multiple stressors and the short time they have to recover. They have already suggested the need to provide psychological and other alternative support, as health workers are often reluctant to seek care for themselves. In addition, several qualitative studies [3, 13, 21] exploring workers' experiences during the pandemic support the importance of understanding workers' psychological realities and providing alternative support as a possible option to reduce future rates of mental illness. To this end, understanding the mental health status of out-of-hospital emergency workers should be the first step in planning interventions to improve psychological resilience within the health care system.

This research is needed to provide information about the opinions, beliefs, and feelings at a particular time and in a particular group of professionals, given the lack of previous qualitative research with out-of-hospital professionals and the high scores on the scales used. We believe that this research would provide valuable data on the behavior and experiences of these professionals in their work during COVID-19.

Based on the results obtained of the previous quantitative study carried out by our research team [4], the need to comprehensive analyse of the experiences of those with high levels of depression from a qualitative perspective was identified. Therefore, the aim of this study it was explore the experiences, coping strategies and influencing factors of Spanish out-of-hospital emergency workers with high levels of depression during the COVID-19 pandemic. It was also assessed the coping strategies and identified the key factors involved in this process, considering that preventive interventions and health promotion strategies could be developed based on these factors.

## Methods

### Aim

To explore the experiences, coping strategies and influencing factors of Spanish out-of-hospital emergency workers with high levels of depression during the COVID-19 pandemic.

### Study design

The approach was to do a phenomenologically oriented study, following the theoretical framework of the humanistic psychologist Carl Rogers [22]. This methodology allows for the elaboration of psychological constructs that must be inferred from the behaviour and social experience of individuals. It helps to understand the essence of the phenomenon, based on how the individual experiences it through their history, and compares it with the experiences of people in similar situations [23]. According to Rogers, a person perceives their experience as reality, and the development of their self-concept from the phenomenological field defines it as that which “*includes everything that is experienced by the body,* (irrespective of) *whether these experiences are sustained by consciousness or not"* [22, 24]*.*

In-deep interviews were used to collect data, as this is the best technique for deepening and visualising individual experiences, and thus, understanding the meaning of such experiences. The study is presented according to the Standards for Reporting Qualitative Research (*COREQ)* checklist [25].

### Participants

A previous quantitative study carried out between February 1 and April 30, 2021 in Spain, showed that 18.2% of workers scored high on the depression scale. The total number of potential participants who were severely depressed was 303 and as this was a voluntary study, everyone who responded was included. Those who requested feedback on their health status assessed in the quantitative study, and who met the inclusion criteria, were invited to participate in this study. Initially, 10 HCWs responded, but later, seven participants (three doctors, one nurse, and three EMTs) volunteered to participate. Thus, seven interviews were conducted in May of 2021; each participant was coded with the letters ‘c’ and ‘l’ followed by an Arabic number (1–7). The interview of the only male participant (cl.2) was not included in the study because he was unable to work at the beginning of the pandemic. The main characteristics of the participants are shown in Table 1. The inclusion criteria were: participation in the previous quantitative study and scored higher than 25 on the depression scale, who volunteered to answer the qualitative study interview, who understood Spanish and could express themselves fluently in Spanish, who had basic computer skills, an Internet connection and agreed to be recorded during the interview, and who were working at the time of the survey. In addition, the exclusion criteria were: having been infected by SARS-CoV-2, suffering from another illness or being pregnant during the response period of the survey that inquired about depressive symptoms, showing high emotional impact throughout the interview that could worsen their mental health.

**Table 1:** Characteristics of the participants

CODE	SEX	AGE	PROFESSIONAL CATEGORY
cl.1	Woman	33	EMT
cl.3	Woman	46	Doctor
cl.4	Woman	44	Doctor
cl.5	Woman	46	Doctor
cl.6	Woman	44	EMT
cl.7	Woman	36	Nurse

*Abbreviations*: Emergency Medical Technician (*EMT*)

### Data confidentiality and ethical assessment

Informed consent was obtained from all subjects. Participants were informed of the ethical principles of confidentiality, protection of personal data and guarantee of digital rights in force in Spain. The study was conducted in accordance with the tenets of the Declaration of Helsinki and was approved by Medical Ethics and Research Committee of the Valladolid East Area (Castilla-León, Spain). Registration PI21-2318. (20 May 2021).

Only the principal investigator had access to the identification and personal data and was allowed to process them.

As the participants were emotionally vulnerable, psychological support was offered to them as part of this project. This support is currently being actively offered to one person.

### Data collection technique

HCWs who agreed to participate were invited by e-mail, explaining the purpose of the study and the need for informed consent to participate. The day and time of the online interviews were arranged.

The interviews lasted between 22 and 75 min and were conducted by six members of the research team. They were all nurses and all women. One of them also has a PhD in nursing and MSc in mental health. Another is a psychologist with a PhD in education). Several interviewers participated because (i) the limited time available the interviewees’ schedules, (ii) to avoid any bias related to a previous relationship between the interviewer and interviewee, and to facilitate free expression and anonymity. In order to make the data collection process consistent, the novel interviewers were trained by simulating an interview. The interviewers were given an orientation script on the topics that might arise and the role of the interviewer as a passive facilitator of the story was emphasised. The script consisted of eight thematic areas, including a) the most shocking experiences at work during the pandemic; (b) feelings and emotions experienced; (c) perception of safety due to the availability of Personal Protective Equipment (PPE); (d) management of the pandemic by the health authorities; (e) relationship with colleagues; (f) impairment of their personal life; (g) effect of vaccination; (h) access to psychological support.

The interviews, whose duration was between 45 and 60 min, were recorded in audio-visual format, with the prior oral and written consent of the participants, using the online platform Jitsi ®. These files were stored in Dropbox® for subsequent literal and systematic transcription. The data obtained were sufficient to achieve the proposed objectives.

At the end of the interviews, they were invited to write a narrative account of their experience, which they could email to the principal investigator.

### Data analysis and triangulation

The researchers ensured the data credibility through deep and long involvement with the data and member checking. After several readings from start to end of each interview, the data were first coded individually by each researcher and then reviewed by the remaining eight members of the research team to reach consensus on the coding. A total of 306 codes were generated and grouped into 14 initial areas, using the ATLAS.ti® v22 software. Thematic analysis was carried out following the method described by Bran and Clarke [26], starting with a reading of the material, generating initial codes that expressed the meaning evoked, and organising them into four thematic groups. The content was then organised into categories and the analysis of the content as a whole was undertaken.

### Rigor

Two researchers with previous experience in qualitative research taught the other researchers how to collect and analyse the data. Highly secure tools with good internet connection were used to conduct the interviews, following the recommendations for qualitative research in times of confinement [27]. In order to ensure the scientific rigour of the study, the guidelines provided by Lincoln and Guba [28] were followed and each step was left to ensure the audit trail. Triangulation was carried out between the researchers and the final categories were decided by the whole team after a two-day meeting, taking into account the available narrative stories. An attitude of constant reflexivity was maintained throughout the process, and the researchers were careful not to let their previous experience of the study bias their decisions. In addition, two researchers who were not involved in the project and who were experts in qualitative methodology reviewed the study. As an exercise in reflexivity, before conducting the interviews and writing the script, each researcher produced a written reflection on the topic in order to be aware of their ideas about their personal experience and to avoid contamination of the data obtained.

Similarly, triangulation of the techniques (quantitative/qualitative), the sources (interview/narrative story), and the researchers was carried out.

## Results

**Fig. 1:**
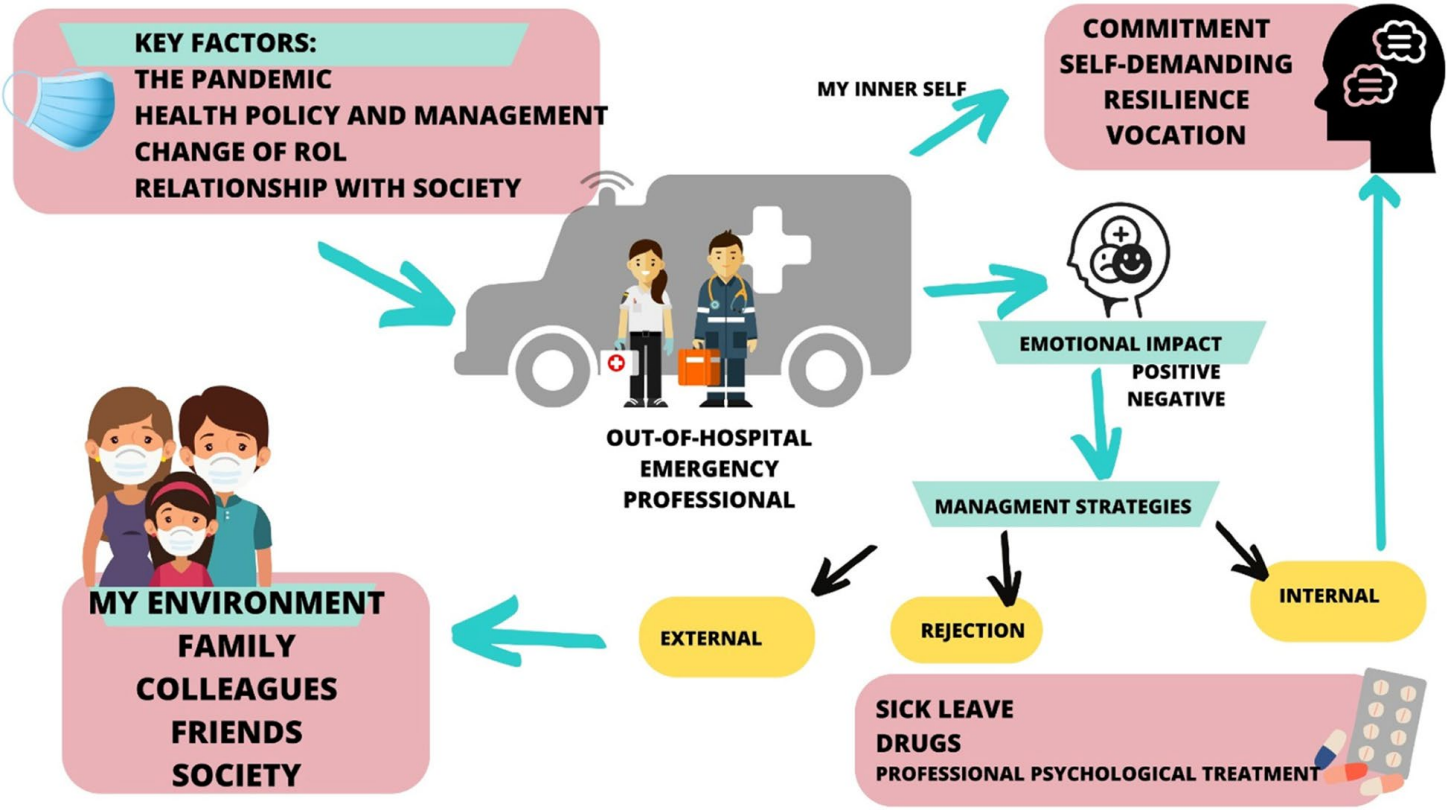
Factors interaction on out-of-hospital emergency HCWs’ mental health

The findings are based on the in-depth analysis of the verbatim transcripts of the interviews and the reflections by the research team, which in some cases drew on the written report. The data were analysed under the central theme of depression among out-of-hospital emergency HCWs and their experiences. Based on the participants’ experiences, the factors that could contribute to an altered mood that could significantly affect their mental health were identified. The factors and their roles are shown in Fig. 1.

### The emotional impact of assuming the professional role: stress and fear* versus* vocation and responsibility

When analysing the meaning of the professional experience of out-of-hospital emergency HCWs, it is necessary to refer to the emotions generated. Participants expressed predominantly negative emotions, including stress, anxiety, sadness, and frustration. Symptoms of depression were described as collapse, overflow, dislocation, and hopelessness.

Being unable to provide more for patients or feeling separated from their homes, unable to return, were some of the reasons for the anger and guilt felt by participants. The constant tension left participants feeling exhausted and unable to disconnect from their work.

*“Then I bring things home, when before I used to leave things at work, everything ... Not now, now I come home, and I go round things and I complain, and I have embittered my boyfriend, poor guy" (cl.7).*

In many cases, they reported being afraid of being infected, of transmitting the disease and even of dying as a result.

On the other hand, some participants described, positive feelings of satisfaction, comfort, cordiality, and gratitude due to the relationship with colleagues. Vocation, commitment, and responsibility motivated the participants to work in such difficult circumstances.

*"But from the very beginning I also offered, with my boss and others, to organise a commission to try to work safely with what we had and, to be able to organise the services. Well, on the one hand, it was good for me because I was able to have something, something active in my head, something to do, to organise” (cl.4).*

### Factors influencing the development of negative emotions

#### The cruelty of the pandemic

During the pandemic, the severity of the situation in terms of the number of cases and the lack of a short-term solution meant that the perception of out-of-hospital emergency HCWs changed over time. The scenario experienced was complicated, especially in the first phase of the pandemic. Participants experienced conflicting feelings, ranging from a high level of uncertainty about the available information and the consequences of the pandemic, to a feeling of strength and ability to resolve critical situations. Nevertheless, they were forced to change the way they worked, having to adapt to the PPE, the frequently changing protocol, and the impact of the disease on their colleagues or themselves.

*"... It was necessary to change the whole way of working together, with the fact that I was in a different (workplace) every day, with new people, and I didn’t know which day I had to work and which day I didn’t; all this made me very tired, especially psychologically." (cl.7)*

The basic Life Support EMTs had the worst conditions, complaining more often that the situation was overwhelming. Over time, everyone assumed that the duration would be longer than expected, leading to experiences of depersonalisation and unreality.

Participants complained of fatigue from caring for while taking care of so many patients affected by the virus and anxiety about not being able to provide the usual care to the person and their family. Participants recalled specific situations that affected them deeply and left them feeling defeated and guilty.

*“Our patients have seen a thousand times on television that when a guy wearing something like a scuba diver appears, you are taken into a small room, where you are connected to a thousand things, absolutely abandoned, unconscious. So, when I pick these people up and I am the one with the scythe, I say, "come on, instead of wearing white, I should wear black" (ironically). You have to explain a lot of things and of course they all have the same expression on their faces, staring at nowhere, like lost, with fear…” (cl.3).*

The vaccine provided reassurance by reducing the risk of infection. It was a turning point that brought hope, but it also made the out-of- hospital emergency HCWs a little angry as the restrictions on the population were relaxed.

#### The helplessness of professionals in relation to health management and policy

One participant felt that the health crisis had been adequately managed in the circumstances, acknowledging the difficulty of the situation for managers. However, the other participants stated that they experienced poor working conditions, a feeling of abandonment, pressure imposed by superiors who treated them rudely, and fear of the consequences of refusal to comply with the requirements.

*«... They abandoned us, mistreated us, undervalued us, and we are still there..." (cl.3)*

The protective equipment was not suited to the working conditions or the size of the worker.

*"Well, we've already adjusted to almost everything. I mean, I'm wearing a suit that’s eight hundred sizes bigger than me. I've learnt how to put it on so that I do not fall and do not miss any safety point" (cl.3)*

There was a difference in EMT between basic and advanced Life Support, with the former being more affected. There was a lack of education and training to provide adequate patient care during the pandemic.

*"But the first time you are alone, and another collage says "here, the patient is yours". Your colleague is driving, and you have to take care of the patient. Then, you go there thinking: "I've just started, I don't know if I'm prepared for certain types of events that may happen" (cl1)*.

The protocols to guide the process arrived late, which exacerbated feelings of insecurity, powerlessness, uncertainty, and ethical dilemma about the decisions that needed to be made.

*"No one had a clue, and we were all learning" (cl3)*.

The out-of-hospital emergency HCWs complained about the lack of support and recognition from their supervisors and the lack of psychological support resources.

*"Well, if they don’t even give you clothes (PPT), how are they going to worry about how you are psychologically... » (cl.6)*

#### Reorganisation of the care and the roles in the emergency response team

The pandemic changed the way of working in the out-of-hospital emergency care was delivered. On many occasions, the unity of the HCW team at work was as the roles of workers were changed by consensus. Early in the pandemic, it was decided that only the doctor and nurse would enter the patient's home for assessment, in order to minimise the number of HCWs exposed.

Patients were cared for according to initial protocols based on pandemic containment measures. This included the urgent transfer of the patients with basic life support care, which reduced the emotional involvement with patients and relatives.

*" I behaved like a machine: arrive, load, and leave. Another one, you arrive, you load, and you leave. I mean, you cried because you cried with them, but you couldn't... you couldn't even see their tears through the glasses, that is how it was. And you could see them being they were torn away from their homes, you know… and they died " (cl6)*

Several participants said that families perceived their attention as "a scythe" tearing their loved ones away.

*"I had to act in a way that made me feel guilty. It was as if you were snatching the life of these people', their family members"." (cl3)*

#### Ambivalence in relation to society

In Spain, society’s gratitude to the HCWs was initially perceived in the applause from the windows and balconies every day at 8 p.m.

*"... At the beginning of the pandemic, everything was "flower power", of "long live to the health workers”, we are all going to get out of this " (cl4)*

Over time, the HCWs began to feel disappointed by the lack of social support and recognition for their hard work, as well as by irresponsible behaviour in society. This led to conflicting feelings and ambivalence among the HCWs, who initially accepted the initial social support, but later rejected the irresponsible behaviour.

*"There are some people who are covid denial, people who are like laughing at this, like the pandemic is a lie” (cl. 1)*

HCWs did not identify with the heroic role assigned to them by society.

### Personal protection through coping strategies

Participants described different ways of coping with the emotional impact of the pandemic and their work.

At the internal level, the HCWs developed self-strategies focused on strengthening resilience to face situations such as the constant changes in protocols, lack of resources, lack of training, job changes, and job instability by signing several contracts in a short period of time.

*"Yes, they are longer contracts, but in bad conditions... He was a bank nurse (without a permanent contract) from June of last year to June of this year". (cl.7)*

However, the ability to anticipate was another common coping behaviour among the respondents, in order to have a sense of control.

*"We have an enormous situation, we have to look for solutions, and we have to try to adapt and position ourselves as well as possible" (cl3).*

In terms of the emotional approach, two strategies were used. One strategy focused on managing negative emotions, such as anger or frustration, by rationalising the fear of the virus, irony, crying, yoga, music therapy, or sport.

*"I try to say, can we do this? Yes, yes, we can do it, great! how nice, and I manage... and without complaining too much..." (cl.3)* (ironic)

The other strategy focused on avoidance, such as sleeping and resting, staying away from family, isolating from the media, or depersonalising at work.

Externally, the HCWs had three types of support, including support from peers, who had similar experiences; support from friends, which in most cases served as emotional relief; and support from family as a way of temporarily escape reality and disconnect from professional life.

Other responses and behaviours of a positive nature were also developed, such as understanding, commitment, altruism, solidarity, and empathy were also developed, which helped to reduce the levels of distress and grief.

*"I think I have more empathy, in general, with all of humanity. I feel everyone's things more" (cl5).*

Most participants did not use psychotropic medication; only one respondent did. One participant sought psychological support but stopped after two sessions. In the midst of high self-demand and responsibilities, these professionals did not consider taking temporary sick leave, as this would have meant seeking specialised treatment and overburdening the rest of the team.

*"I did not take sick leave for my team members. I could not leave my partner (work colleague) to face this alone..." (cl.6)*

### Good practices for the future

The final part of this study consisted of suggestions for improving and changing the model of health management model and the dynamics of psychosocial risk prevention, based on what the participants had said.

Management by decision makers should be proposed based on their position and responsibility. Management decisions should be made by the manager, without delegating this task to the emergency HCW. The development of care protocols is the responsibility of central units, which should be careful not to overinform the workers. Managers must ensure that workers are trained in its correct use.

In such a demanding situation for HCWs, it would be appropriate for their immediate superiors to recognise the work carried out and the effort made to complete the tasks. Also, the exceptional measures that were taken during the pandemic, such as continuous changes in working conditions, including modifications in working hours and changes in the units, should not be normalised, or continued over time.

## Discussion

The cruelty of the challenges they faced during the pandemic, the sense of helplessness they experienced, coupled with the overstretch of society, had a direct impact on the mental health of out-of-hospital emergency HCWs. In such circumstances, the personal strategies and skills were inadequate, making it difficult for them to recognise the need for support to improve their mental health. Several elements were identified that could be improved in the event of another pandemic arises or similar stressful event.

In this study, the out-of-hospital emergency HCWs who had high levels of depression one year after the pandemic were exposed to stressors similar to those experienced by other health workers, such as those in the hospital setting; those who were exposed to a large number of deaths were the most affected [7, 15, 19, 29, 30]. Frontline HCWs experienced fear and anxiety at the onset of the pandemic [31]; specifically, they were afraid of infecting their loved ones or becoming infected themselves, and anxious about the uncertainty of facing an unknown enemy, which led to isolation behaviours [12]. Over time, the prolonged tension turned into long-term exhaustion, sleep problems, and feelings of defeat [31–33]. Participants in this study sometimes voluntarily isolated themselves as a psychological coping strategy, but in other countries, the decrease in social interaction was a consequence of professional stigma [16, 34]. In addition, those who witnessed many deaths, such as the professionals in Canada were particularly unprepared and deeply affected [35].

A common denominator in the studies conducted was widespread dissatisfaction with health management and policies. The lack of institutional support and ineffective communication by managers resulted in unbalance workloads, poor organisation, inefficient inter-sectoral relations, contradictory decisions, ineffective distribution of human resources, lack of training, lack of integrated health protocols, high staff turnover and lack of specialised professionals [36]. These factors, in some cases, led to a change in the role of workers with changes in the dynamics of the team [37]. In addition to organisational challenges, legal ambivalence led to ethical conflicts [36]. In Canada, the managers were present and responsive, transparent in their communication, and involved HCWs in the development and practice of policies and procedures [35]. This protected the HCWs from burnout and ensured the long-term sustainability of the workforce, something that was missing in most Spanish emergency services.

The relationship of out-of-hospital emergency HCWs with society has gone through different stages. Initially, emergency personnel were seen an indispensable and applauded for their hard work, except on certain occasions when they were stigmatised [31, 38, 39]. Over time, feelings of disappointment with society increased, given the lack of solidarity, which increased the suffering among HCWs and led to feelings of frustration and helplessness [34]. This kind of duality also occurred with social networks, considering that they provided support and allowed communication with their professional colleagues and the closest people, and they also generated excessive information about changing protocols that was impossible to process [30].

To improve the situation caused by the pandemic, HCWs tried to adapt by anticipating possible consequences, and seeking coping strategies such as social support from collage, talking with therapist or close others, using humour and exercising [39, 40]. They sought support from their colleagues, as they were all in the same situation, which provided them relief and a sense of security [39]. Subsequently, the HCWs who shared their experiences were friends and family, with whom physical distance was maintained to try to protect them from contagion [31, 38, 39]. Other studies have also used adherence to values, religion-centred coping, community service, and altruism [21, 40, 41].

The results showed the difficulty in recognising that they were suffering from a change in the mental state and the rejection of psychotropic medication and psychological intervention, an aspect that was highlighted by Pollock et al. [40]. Participants did not seek psychological help because they assumed they had sufficient tools to develop resilience and skills to withstand high levels of stress. Their work was overly self-demanding, which reduced the effectiveness of their strategies [41, 42]. Many of them were unaware of the deterioration in their mental health until they participated in the previous quantitative study.

In terms of suggestions for improvement, most studies focused on health policies to prevent mental deterioration in frontline workers and explored ways to improve management as a whole; these aspects have been recommended in other previous health crises [40]. Actions taken by the Health Services have had a negative impact on the mental health of out-of-hospital HCWs, such as poor planning in resource management or the lack of institutional support [36, 40, 43]. Failure to support is not provided to HCWs vulnerable to emergencies can lead to psychological distress, which can become chronic [39, 44]. Based on participants’ experiences and a literature review, the incidence of these disorders could be reduced by suggesting several improvements, such as having sufficient material resources, having clear protocols and avoiding over-information, promoting job stability and care of the emergency HCWs by institutions, having telemedicine services attended by professionals in psychology and psychiatry, creating online support groups consisting of like-minded health care workers, monitoring vulnerable groups and providing continuous individualised services continuously, and providing learning opportunities for emotional management and promoting HCM the resilience [6, 39, 41].

## Conclusions

The experiences of the out-of-hospital emergency HCWs during the pandemic have had a major emotional impact, and their coping strategies have sometimes been ineffective, leading to depressive states in some of them.

These individuals need to be offered help. Ways of identifying to recognise problems need to be urgently identified. A balance between self-demand and professional responsibilities needs to be maintained to avoid feelings of frustration and guilt.

The COVID-19 pandemic highlighted the need to improve health crisis management policies. It also demonstrated the need to instil a culture of solidarity with the HCWs in society.

### Methodological limitations and difficulties

The number of participants was small but considering that the participants who shared their experiences were emotionally highly vulnerable, their contributions are quite relevant. The richness of the data allowed us to achieve the initial aim.

The variability of the sample was limited by gender, as all participants were women.

Although the results are not intended to be generalisable, they are transferable to similar contexts.

While planning the study design, virtual interviews were identified as the best option for data collection. Face-to-face interviews were complicated by pandemic-related restrictions and the geographical distance between the interviewers and respondents. Although virtual interaction is different from face-to-face interaction, the problems of physical distance, and the inherent difficulties could be reduced through the empathy of the interviewers. As the interviews were conducted among peers, it was not necessary to explain some concepts and situations in detail as all interviewers, and interviewees understood the context very well. In order to avoid the results being the researches’ vision and to achieve neutrality, we carried out the reflectivity and the review by a member of the team who does not work in the same environment.

The interviews were conducted by several interviewers in order to match the timing of the interview with the interviewee's preferences. Although having several interviewers may be a limitation, they had several meetings to agree on a common interview script.

Only those people with high levels of depression who requested feedback on their scores on the scales of the quantitative study and who generated an identification code were included in the study. This means that many candidates who probably met the criteria for inclusion in the qualitative study could not be contacted. The main reason why we did not contact those who were interested in knowing their mental health status and who had high scores for depression was that they generated the wrong code.

The data collection process was triangulated by a free-form narrative account submitted by the participants as a reflection on their experience. These accounts were referred to during dilemmas in the research team about the discourse of the interview of some participants’ interviews, but they were not analysed in depth through coding and categorisation.

### Implications for clinical practice

The mental health care of out-of-hospital emergency HCWs is summarised by the need to care for and protect health care workers who care for others. A working culture should be promoted that does not stigmatise people and normalises mental health problems in the community of emergency HCWs should be promoted. Psychological support services for work-related situations are also desirable. Teamwork and good treatment among colleagues should be encouraged. Peer support groups led by mental health professionals should be established. Programmes to prevent burnout and increase self-efficacy should be implemented. Ongoing training programmes should be provided to teach effective coping strategies and anxiety management in adverse work situations.

### Supplementary Information

**Additional file 1.**

## Data Availability

The datasets used and/or analysed during the current study are available from the corresponding author on reasonable request.

## Abbreviations

DASS-21: Depression, Anxiety, and Stress Scale
COVID: Coronavirus Disease
RNA: Ribonucleic Acid
SAR-COV: Severe Acute Respiratory Syndrome Coronavirus
WHO: World Health Organisation
HCWs: Health Care Workers
COREQ: Consolidated Criteria for Reporting Qualitative Research
EMTs: Emergency Medical Technicians
PhD: Doctor of Philosophy
MSc: Master of Science
PPE: Personal Protective Equipment
ATLAS.ti: Computer-assisted qualitative data analysis software
