# Supplementary material for: Experiences of Spanish out-of-hospital emergency workers with high levels of depression during the COVID-19 pandemic: a qualitative study
Source: Arch Public Health. 2024 Jan 30;82:15. doi: 10.1186/s13690-023-01233-w (PMC10826038; doi:10.1186/s13690-023-01233-w)

**Appendix 1:**

**Script developed for conducting the semi-structured interviews**

All research agrees on a semi-structured interview as an orientation guide.

FIRST CONTACT WITH Jitsi®: (maximum 5 min.)

- Introducing the researcher.

- Give information about the study.

- Insist on anonymity and confidentiality.

- Clarify any doubts.

- Thank them for their participation.

- Ask for informed consent to participate in the study and permission to record the session in Jitsi®.

- Ask for permission to use their name.

BEGINNING THE INTERVIEW: (5 min)

- Introduce the topic: talk about the early stages of the pandemic, the interviewer's personal experiences on a personal and professional level.

- Be open to the possibility of having sequelae such as anxiety and depression in order to empathise with the interviewee.

- We are not here to judge, all the emotions we have experienced during the pandemic are legitimate and anything can happen to us in this life.

OPEN QUESTIONS: (25-40 min)

Three moments: now, during the pandemic, at the beginning of the pandemic.

Factors that you think have led you to be like this. Help to organise by focusing only on work-related factors: how was the management in your department during the pandemic, was there any support from colleagues, was there any opportunity for psychological support during the pandemic.

Now (briefly)

I would like to ask you how you feel right now?

During (at the beginning and at the end, as parts within this year)

1. Do you think that working as a health worker in the pandemic has affected the way you deal with your daily work? How has it done so? How has your work environment affected your personal and family life during the pandemic?

2. What aspects of your work have most affected you during the pandemic?

3. How did you feel at work during the worst moments of the pandemic?

4. Were you afraid at work during the pandemic? If so, are you still afraid?

5. Did you feel unsafe wearing PPE at work during the early stages of the pandemic?

6. Do you feel safer at work now that you have been vaccinated?

7. Do you feel you were adequately trained to deal with the pandemic? What were your strengths at work during the pandemic? What were your weaknesses?

8. How would you rate the management of the pandemic in your health service or company? Do you think there are things that could have been better organised at management level in your work? What things? Who would be responsible for these actions?

9. Do you think you could have done more in your work during the worst moments of the pandemic? If so, what could you have done?

10. Do you think the pandemic affected your relationship with colleagues? If so, in what way?

11. Can you give me some words that could define how you felt at the time?

- Did you feel anger at work during the pandemic? If so, about what, and do you feel it now? If so, about what?
- Did you feel sad during the pandemic? What symptoms did you have? At what times did the sadness occur?
- Did you experience anxiety during the pandemic? What symptoms did you have? At what times did the anxiety occur?

12. Did you receive any psychological support, and if so, what kind of support, and did you need any pharmacological treatment?

Before the pandemic

1. Did you suffer from anxiety and/or depression before the pandemic? If so, did you need medication or counselling?

Currently

2. What has changed in you as a health care worker since the pandemic?

3. What do you think you need to feel better now?

END OF INTERVIEW: (10 min)

- Make a general summary of the interview to confirm that what the interviewee wanted to show was understood by the interviewer.

- Ask for any information that the interviewee feels is important and has not been covered.

- Thank them for their time and help.

- Offer psychological support.

**Appendix 2:**

**Resolution of the Ethical Committee of the Quantitative Study**


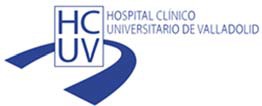

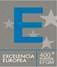

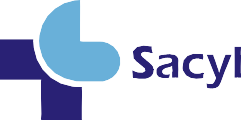


Avda. Ramón y Cajal, 3 - 47003 Valladolid Tel.: 983 42 00 00 - Fax 983 25 75 11

[gerente.hcuv@saludcastillayleon.es](mailto:gerente.hcuv@saludcastillayleon.es)

COMITÉ DE ÉTICA DE LA INVESTIGACIÓN CON MEDICAMENTOS ÁREA DE SALUD VALLADOLID

Valladolid a 14 de enero de 2021

En la reunión del CEIm ÁREA DE SALUD VALLADOLID ESTE del 14 de enero de 2021, se pro- cedió a la evaluación de los aspectos éticos del siguiente proyecto de investigación.

|  |  | I.P.: SUSANA NAVALPOTRO PASCUAL |
| --- | --- | --- |
|  |  | EQUIPO: BEJARANO RAMÍREZ, JUAN FRAN- |
|  |  | CISCO; CÁRDABA GARCÍA, ROSA MARÍA; |
|  |  | CASTEJÓN DE LA ENCINA, MARÍA ELENA; |
|  |  | FERNÁN PÉREZ, PATRICIA; FERNÁNDEZ DO- |
|  |  | MÍNGUEZ, JUAN JOSÉ; GARCÍA SANTA BASÍ- |
| PI- 20-  2052  COVID | PREVALENCIA DE ANSIEDAD, ESTRÉS, DEPRESIÓN Y AUTOEFICACIA EN LOS PROFE- SIONALES DE LOS SERVICIOS DE EMERGENCIAS ESPAÑOLES DURANTE LA PANDEMIA DE LA COVID 19  (IMPSYCOVID19-RINVEMER) | LEA NOEMÍ; JIMÉNEZ ALEGRE, JULIO; MAR- TÍN SÁNCHEZ, RAFAEL; MARTÍNEZ CABALLERO CARMEN MARÍA; MERINO RE- GUERA, BEATRIZ; MIGUEL SALDAÑA, FER- NANDO; MORALES SÁNCHEZ, ALMUDENA; NAVALPOTRO PASCUAL, JOSÉ MARÍA; ONRU- BIA BATICÓN, HENAR; PASTOR BENITO,  ELENA; POLO PORTES, CARLOS EDUARDO; SÁNCHEZ DEL RIO, LETICIA; SOTO CÁMARA, |
|  |  | RAÚL; MATELLAN; HERNÁNDEZ, MARÍA PAZ; |
|  |  | MOLINA OLIVA, MARÍA; MOYA RODRÍGUEZ- |
|  |  | CARRETERO, MARTA; REQUES MARUGAN ANA |
|  |  | MARÍA. |
|  |  | URGENCIAS Y EMERGENCIAS PREHOSPITALA- |
|  |  | RIAS |

A continuación, les señalo los acuerdos tomados por el CEIm ÁREA DE SALUD VALLADOLID ESTE en relación a dicho Proyecto de Investigación:

Considerando que el Proyecto contempla los Convenios y Normas establecidos en la legislación española en el ámbito de la investigación biomédica, la protección de datos de carácter perso- nal y la bioética, se hace constar el **informe favorable** y la **aceptación** del Comité de Ética de la Investigación con Medicamentos Área de Salud Valladolid Este.

Un cordial saludo.


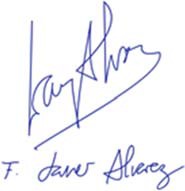


Dr. F. Javier Álvarez.

CEIm Área de Salud Valladolid Este Hospital Clínico Universitario de Valladolid Farmacología, Facultad de Medicina, Universidad de Valladolid,

c/ Ramón y Cajal 7,47005 Valladolid

[alvarez@med.uva.es,](mailto:alvarez@med.uva.es) [jalvarezgo@saludcastillayleon.es tel.:](mailto:jalvarezgo@saludcastillayleon.estel) 983 423077


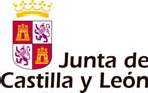


**Appendix 3:**

**Resolution of the Ethical Committee of the Qualitative Study**


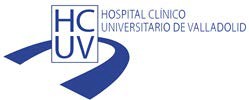

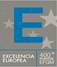


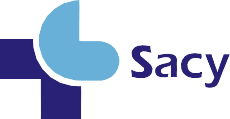


Avda. Ramón y Cajal, 3 - 47003 Valladolid Tel.: 983 42 00 00 - Fax 983 25 75 11

[gerente.hcuv@saludcastillayleon.es](mailto:gerente.hcuv@saludcastillayleon.es)

COMITÉ DE ÉTICA DE LA INVESTIGACIÓN CON MEDICAMENTOS ÁREA DE SALUD VALLADOLID

Valladolid a 20 de mayo de 2021

En la reunión del CEIm ÁREA DE SALUD VALLADOLID ESTE del 20 de mayo de 2021, se procedió a la evaluación de los aspectos éticos del siguiente proyecto de investi- gación.

| PI 21-2318 NO HCUV | APROXIMACIÓN CUALITATIVA A LA EXPERIENCIA DE LOS PROFESIO- NALES DE EXTRAHOSPITALARIA CON ALTOS NIVELES DE DEPRE- SIÓN DURANTE LA PANDEMIA DE LA COVID-19  (IMPSYCOVID19-RINVEMER) | I.P.: SUSANA NAVALPOTRO EQUIPO: JUAN BEJARANO RAMÍREZ, ROSA MARÍA CÁRDABA GARCÍA, MARÍA ELENA CASTEJÓN DE LA EN- CINA, PATRICIA FERNÁN PÉ- REZ, JUAN JOSÉ FERNÁNDEZ DOMÍNGUEZ,  NOEMÍ GARCÍA SANTA BASI- LIA, JULIO JIMÉNEZ ALEGRE, RAFAEL MARTÍN SÁNCHEZ, CARMEN MARTÍNEZ CABA- LLERO, MARÍA PAZ MATE- LLÁN HERNÁNDEZ, BEATRIZ MERINO REGUERA, FERNANDO MIGUEL SAL- DAÑA, MARÍA MOLINA OLIVA, ALMUDENA MORALES SÁN- CHEZ, MARTA MOYA RODRÍ- GUEZ-CARRETERO, JOSE MA- RÍA NAVALPOTRO PASCUAL, HENAR ONRUBIA BATICÓN, ELENA PASTOR BENITO, CAR- LOS EDUARDO POLO POR- TES, LETICIA SÁNCHEZ DEL RIO, ANA MARÍA REQUÉS MARUGÁN, RAÚL SOTO CÁ- MARA  URGENCIAS Y EMERGENCIAS PREHOSPITALARIAS |
| --- | --- | --- |

A continuación, les señalo los acuerdos tomados por el CEIm ÁREA DE SALUD VA- LLADOLID ESTE en relación a dicho Proyecto de Investigación:


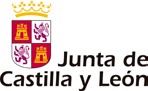


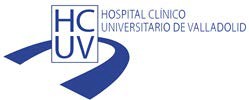

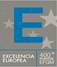


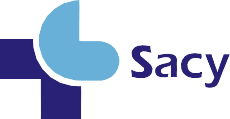


Avda. Ramón y Cajal, 3 - 47003 Valladolid Tel.: 983 42 00 00 - Fax 983 25 75 11

[gerente.hcuv@saludcastillayleon.es](mailto:gerente.hcuv@saludcastillayleon.es)

Considerando que el Proyecto contempla los Convenios y Normas establecidos en la legislación española en el ámbito de la investigación biomédica, la protección de da- tos de carácter personal y la bioética, se hace constar el **informe favorable** y la **aceptación** del Comité de Ética de la Investigación con Medicamentos Área de Salud Valladolid Este.

Un cordial saludo.


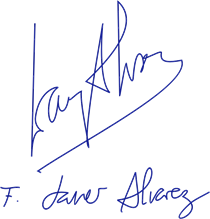


Dr. F. Javier Álvarez.

CEIm Área de Salud Valladolid Este Hospital Clínico Universitario de Valladolid Farmacología, Facultad de Medicina, Universidad de Valladolid,

c/ Ramón y Cajal 7,47005 Valladolid [alvarez@med.uva.es](mailto:alvarez@med.uva.es), [jalvarezgo@saludcastillayleon.es](mailto:jalvarezgo@saludcastillayleon.es) tel.: 983 423077


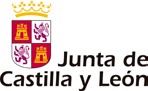

Supplement: Supplementary file 1 — Additional file 1. [file 13690_2023_1233_MOESM1_ESM.docx]
